# Sclero-Hyalinized Low-Grade Appendiceal Mucinous Neoplasm Clinically Mimicking an Ovarian Mass

**DOI:** 10.1155/2022/9404615

**Published:** 2022-07-15

**Authors:** Nnaemeka Thaddeus Onyishi, Anthony Jude Edeh, Ngozi Regina Dim

**Affiliations:** ^1^Department of Histopathology, Enugu State University College of Medicine Parklane Enugu, Nigeria; ^2^Department of Pathology, University of Sierra Leone Teaching Hospital Complex Freetown, Sierra Leone; ^3^Surgery Department, Enugu State University College of Medicine Parklane, Enugu, Nigeria; ^4^Department of Radiation Medicine, College of Medicine University of Nigeria, Ituku Ozalla Campus, Nigeria

## Abstract

Low-grade appendiceal mucinous neoplasm is a tumor of the appendix whose major clinical relevance derives from its inherent potential for peritoneal dissemination as pseudomyxoma peritonei. It sometimes poses challenges in clinical, radiological, and pathological diagnosis, and it may be confused with gynecological conditions in females. We report a case of low-grade appendiceal mucinous neoplasm presenting as firm sclerotic cystic mass and was initially suspected to be an ovarian mass in a postmenopausal woman. We review the literature for the pathogenesis and clinical implication of sclerohyalinization in mucinous appendiceal tumors.

## 1. Introduction

Neoplasms of the vermiform appendix are rare, estimated to occur in 2% percent of all appendectomy tissues [1]. Primary epithelial neoplasm is present in about 0.6% of all appendectomies just as mucinous neoplasm is estimated to occur in 0.3% of all surgically resected appendices [2]. Low-grade appendiceal mucinous neoplasm (LAMN) is a terminology originally introduced by Misdraji and colleagues [3]. The term is used in the current classification schemes to denote mucinous appendiceal tumors with low-grade cytology in addition to any of the following: loss of the lamina propria and muscularis mucosae, fibrosis of the submucosa, pushing growth into the wall of the appendix, dissecting acellular mucin in the wall of the appendix, and cellular or acellular mucin outside of the appendix [4, 5]. The clinical importance of LAMN lies in its ability to disseminate throughout the peritoneal cavity as pseudomyxoma peritonei (PMP), a condition that often runs an interminable clinical course requiring aggressive treatment. About 20% of patients with mucinous epithelial neoplasm of the appendix develop PMP [2]. In terms of clinical presentation and pathologic appearance, the majority of LAMN present as abdominal mass or pain with fewer numbers discovered incidentally during procedures for some other conditions. Gross pathology appearances have been described as dilated or cystic in most reported cases [3]. We report a case of cystic sclerohyalinized LAMN mimicking an ovarian mass, and we review the literature for the underlying pathogenetic mechanism and clinical implication of this striking morphology.

## 2. Case Presentation

A 67-year-old African woman, 20 years postmenopausal, and on treatment for diabetes and hypertension, presented with a right lower abdominal swelling of five-month duration. The swelling was of gradual growth with intermittent dull ache that preceded the swelling by a few months. On physical examination, her vital signs were normal. Abdominal palpation revealed a mobile, nontender, soft right lower abdominal mass. A provisional diagnosis of right iliac fossa mass probably of ovarian origin was made prompting a review by a gynecologist. Transabdominal ultrasound scan showed a large, irregular shaped, complex heteroechoic mass, that abuts the right aspect of the urinary bladder and the uterus. The mass had a well-defined, relatively hyperechogenic thick margin and measured 15 × 7.17 × 10.5 cm. Computed tomography (CT) scan was requested for, but the patient could not afford it. She was transferred to the general surgery unit where she underwent exploratory laparotomy.

The finding at laparotomy was a large caecal mass which was adherent to the uterus and bladder. A right hemicolectomy and ileo-transverse 2-layer anastomosis was done with good outcome. She has remained in good health 2 years post-surgery.

**Figure 1:**
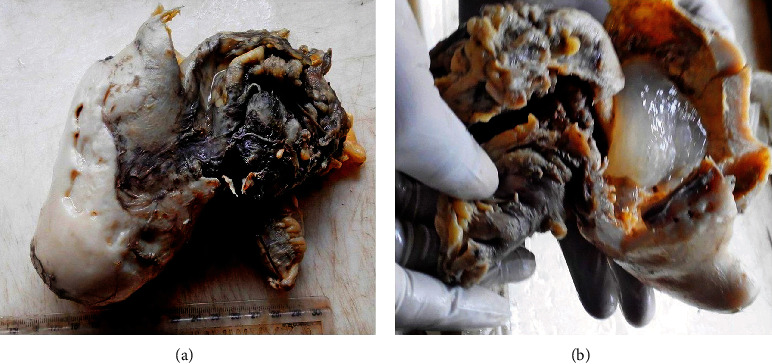
Gross photograph of low-grade appendiceal mucinous neoplasm. (a) It shows formalin-fixed right hemicolectomy tissue consisting of segments of terminal ileum, ascending colon and firm cystic sclero-hyalinized appendiceal mucinous neoplasm. (b) The cut section of the appendiceal neoplasm hand-held to display the content of gelatinous mucin and the remarkably thick sclerotic wall.

Pathology: Gross examination showed a right hemicolectomy tissue. The appendix was transformed into a firm whitish cystic mass measuring 8 cm × 7 cm × 7 cm. The cyst contained gelatinous mucin, and its wall measured 1 cm in thickness (Figure 1). No perforation was seen, and no mucin deposits were present on the appendix or intestinal tissues.

**Figure 2:**
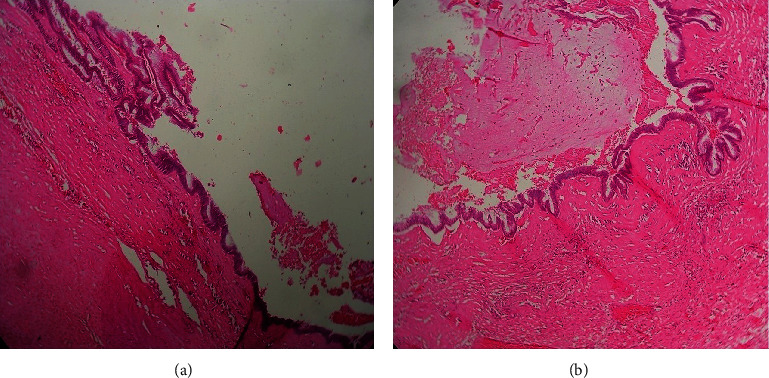
Photomicrograph of low-grade appendiceal mucinous neoplasm. (a) It shows a single layer of neoplastic mucin secreting epithelium resting on a fibrotic wall. (b) It shows pushing invasion into the fibrotic wall and luminal mucin. H&E. ×20 objective.

Histologic sections of the appendix showed mainly intense sclerosis and hyalinizing fibrosis with the mucosal or epithelial tissue denuded in most sections. The lumen showed adherent mucin. Extensive sampling of the mass revealed foci of flat single layer of neoplastic epithelium resting on a fibrotic wall. The epithelial cells were dysplastic with some showing cytoplasmic mucin. The layer of neoplastic epithelium showed outpouchings into the fibrous wall, but no areas of destructive invasion were seen Figure 2. The fibrous wall also had foci of chronic inflammation and calcification (not shown).

## 3. Discussion

Review of existing literature on appendiceal neoplasm portrayed some controversy which revolved around what should be the right taxonomy for mucinous neoplasms, a group of neoplasm that often showed a wide range of clinical behavior varying from indolent to aggressive [3, 4]. Different workers proposed various classification systems and terminologies, but none of the proposed systems could fully reconcile the bland histology exhibited by the tumors with their capacity for disseminated peritoneal disease, PMP. However, there seems to be a convergence towards consensus with recent publication of classification systems, which remarkably improved on past efforts [5]. In the recent classification schemes, LAMN is recognized as appendiceal tumor with flat or undulating epithelium, low-grade nuclear atypia, atrophy of appendiceal lymphoid tissue, and loss of muscularis mucosae and submucosal fibrosis [4]. The present report is a case of LAMN in a 67-year-old postmenopausal woman. Previous reports indicated that 70 to 80 percent of LAMN occurred in women and in 16 to 89 years age range [3, 6]. In terms of clinical presentation, they present either as any or a combination of abdominal pain, abdominal mass, abdominal distension, and acute appendicitis. Some come to attention as incidental finding during unrelated procedures [3, 6].  Table 1 shows the frequency of key morphologic features of mucinous appendiceal neoplasm as described in two different series aggregating to 233 cases.

The present case was cystic with thick hyalinized wall and was adherent to adjacent organs. Long-standing cases of LAMN can grow to a massive size of up to 29 cm with dense fibrous adhesion to surrounding structures [7]. The present case also had foci of inflammation and luminal mucin. Mucinous epithelium, mural fibrosis, and dissecting mucin also seen in this case define LAMN generally and are key components of its diagnostic criteria [5].

Low-grade mucinous neoplasms, having little capacity for deep or widespread visceral invasion, produce dire clinical consequences through copious mucin secretion. MUC2 and MUC5AC genes responsible for secretion and deposition of gel-forming mucin are strongly expressed in all mucinous tumors of the appendix and most PMP irrespective of nuclear grade [8]. Mucin to epithelial cell ratio of between 10 : 1 and 1000: 1 is found in PMP [8]. Excessive accumulation of gelatinous mucin in the peritoneal cavity not only causes increased intraabdominal pressure and compression of visceral organs, but also promotes inflammation and fibrosis with bowel obstruction [9]. Histologic evaluation of peritoneal mucin deposits often showed inflammation, neovascularization, and granulation tissue response which may progress to fibrosis [10]. Misdraji and colleagues found the stroma surrounding extra-appendiceal mucin pools to be “invariably fibrotic or hyalinized and sometimes chronically inflamed” [3]. It is plausible that a similar reaction to percolating mucin in the wall of the appendix causes the fibrotic obliteration of the submucosa and mural hyalinization as was seen in this case suggesting that tissue reaction to mucin may explain the sclerosis seen in LAMN. No study, to the best of our knowledge, has examined the relationship between hyalinization and appendiceal rupture. However, some studies have indicated that the size of appendiceal lesions, when >6 cm, is more likely to be malignant and is associated with increased risk of rupture [11, 12]. Our patient had a grossly enlarged mass, with no perforation or mucin deposits. It is possible that complete and thick capsule-like mural hyalinization could potentially impede rupture and prevent extra-appendiceal spread of LAMN.

Imaging plays a role in the pre-surgical diagnosis and evaluation of LAMN, and aids in follow up of patients for possible complications, such as PMP. Modalities used include ultrasonography, computed tomography (CT), and magnetic resonance imaging (MRI). The characteristic well-demarcated appearance seen at CT imaging of LAMN has been ascribed to high incidence of fibrosis and mural hyalinization [13].

## 4. Conclusion

Low-grade appendiceal mucinous neoplasm has prominent fibrogenic potencies. It sometimes poses challenges in clinical, radiological, and pathological diagnosis; it may be confused with gynecological conditions in females, while some cystic fibrous tumors require extensive sampling before the characteristic neoplastic epithelium can be identified.

## Figures and Tables

**Table 1:** Reported morphologic appearance of LAMN. Features are extracted from two case series [3, 6].

	Frequency
*Gross feature (161 appendices)*	
Grossly unremarkable	7
Dilated or cystic	116
Thick walled	19
Gross rupture	42
Serosal mucin	40
Diverticular disease	6
*Microscopic feature (223 appendices)*	
Diverticular disease	16
Submucosal fibrosis/lymphoid atrophy	136
Mural hyalinization	21
Calcification	66
Atypical mucinous epithelial cells	All cases

## Data Availability

The datasets reported herein are available from the corresponding author on request.
